# Recovery at 30: Integrating Lived Experience Expertise into Mental Health Research in Israel

**DOI:** 10.1007/s10597-024-01369-1

**Published:** 2025-01-06

**Authors:** Shira Alfia-Burstein, Avi Oren, Yael Goldfarb, Renana Stengar-Elran, Vanessa Pinfold, David Roe, Galia S. Moran

**Affiliations:** 1 The Lishma Association for the Integration and Empowerment of People Coping With Mental Health Issues, Hadera, Israel; 2 Psychiatric Rehabilitation Services, Central District, Ramla, Israel; 3 Department of Social Work, Ben Gurion University of the Negev, Be’er Sheva, Israel; 4 Yozma Derech Halev (Initiative from the Heart) – Employment Support for Persons with Lived Experiences of Mental Illnesses, Kfar Saba, Israel; 5 The McPin Foundation, London, UK; 6 Department of Community Mental Health, University of Haifa, Haifa, Israel

**Keywords:** Recovery, Lived experience, Mental health research, Israel, System transformation

## Abstract

A major component of recovery is the inclusion of lived experience to transform the culture of Mental Health (MH) services. In Israel lived experience has been increasingly integrated into services through peer roles. However, lived experience knowledge and expertise has not been sufficiently nor systematically integrated into the design of mental health research. This paper documents an attempt to initiate change by convening multiple stakeholders (with and without lived experience) in a specialized workshop aiming to learn and discuss the potential role of lived experience for mental health research in Israel. Participants raised ideas and core questions on how lived experience can shape research and augment mental health practices and policies. They highlighted current challenges regarding self-disclosure facing lived experience researchers, as well as challenges for developing participatory research collaborations among consumers, family members and practitioners. By bringing to the fore-front the ‘insider perspective’ of MH system as experienced among service users and families, we expect a development of a research culture with reduced paternalism, increased coproduction and recovery-orientation. We hope this endeavor will inspire others and help develop a lived experience expertise-based research network of interested stakeholders.

## Background

Over the last two decades Israel has undergone a transformative shift in its approach to mental health, embracing the vision of recovery. This includes the implementation of psychiatric rehabilitation practices to help service users acquire skills and supports in the community (Aviram, et al, 2012; Roe et al., 2012). This evolution aligns with Anthony’s (1993) vision of recovery, symbolizing a departure from symptom and deficit-centric approaches to a person-centered self-determined perspective. This shift was a result of accumulated and diverse efforts made in Israel since the 1980s’. Then, many citizens with diverse ethno-backgrounds were inflicted with emotional pain related to the aftermaths of immigrations, Holocaust traumas, war and terror. At that time the mental health services were lacking of manpower and resources were depleted (Moran, 2018). In face of this reality, overtime pioneers of the Israeli consumer movement, along with family members, began voicing their frustrations and needs regarding the MH system. In a collective effort involving people with lived experience (service users and family members) along with practitioners, one parliament member and various legislators, Israel achieved the enactment of the Rehabilitation in the Community of Persons with Mental Disabilities Law in 2000 (Aviram, et al, 2012). This significant milestone legislation embraced rehabilitation principles and community integration with impressive outcomes for mental health practice, research and policy. It laid the foundation for the development of numerous recovery-oriented services (Roe et al., 2012), community initiatives and extensive research addressing: law implementation (Aviram et al., 2023; Drake et al., 2011); intervention effectiveness (Hasson-Ohayon et al., 2007; Levy-Frank et al., 2011; Roe et al., 2014); various rehabilitation models (Gelkopf et al., 2016; Hornik-Lurie et al., 2018; Moran et al., 2014, 2017), routine outcome monitoring (Roe et al., 2015a; 2015b; 2022) and cultural adaptation for Arab speaking communities (Daass-Iraqi et al., 2021).

Over the past decades, recognition of the value of knowledge from lived experience to support recovery grew in Israel. Experiential knowledge (“having been there and learnt from the experience”) and mutual support (a reciprocal two-way helping relationship) are gradually acknowledged as essential in balancing hierarchical-professional relationships to a more person-centered self-driven and mutual/co-productive cultures. With this recognition, development and emergence of peer support roles based on lived experience (LE) expertise evolved. As a result, persons with LE can now engage in multiple types of peer-roles across the MH system in Israel. These include consumer-providers in rehabilitation services, peer specialists in psychiatric wards, peer surveyors of community mental health services, peer advocates for rights and more (Goldfarb et al., 2024; Grundman et al., 2021). Additionally, some academic institutions in Israel also embrace LE as a valuable source of knowledge for students (Kraus and Moran et al., 2019; 2023a; 2023b). Yet, the incorporation of LE via research has been more limited.

Consumer involvement in research has been growing in different parts of the world (Banfield et al., 2018; Happell & Roper, 2007). In a growing number of countries there are efforts and progress in developing different approaches and embedding critical thinking to challenge mainstream psychiatric research such as survivor research (Rose, 2021) and mad studies (Beresford & Rose, 2023). Examples also include service user-led research (Pagdon & Jones, 2023), peer research (Guta et al., 2013) and public and patient involvement in research (Richmond et al., 2023). In the UK, research funders require the beneficiaries of health research to be actively involved as partners in research (NIHR, 2019). Unfortunately, in Israel involvement of LE expertise in research is rarer, less formal and more sporadic. Its role as an integral and valid source of knowledge for the transformation of practices and policies has had only marginal influence on services and system design (Moran, 2017, 2018).

This paper will focus on an initiative to set up a research network in Israel including LE experts, embracing a self-determined (“nothing about us without us”) citizen-based and a coproduction approach. The research network aims to create a more balanced integration of LE expertise input to influence practices and policies. For this purpose, we invited mental health stakeholders (with and without LE) to a workshop seminar. The seminar was led by Dr. Vanessa Pinfold, of the McPin foundation, a senior researcher whose organization conducts LE guided research in community mental health care (https://mcpin.org/). Dr. Pinfold has 25 years of experience as a researcher dedicated to carrying out coproduction-research initiatives (e.g. Frerichs et al., 2020; Liberati et al., 2021; Mackay et al., 2022; Sweet et al., 2018). Over the last 10 years she has established and led the McPin Foundation as a research charity designed to champion LE led research in community mental health care.

Here, we describe the workshop which aimed at sparking conversations and driving forward an agenda to develop LE involvement and leadership in research in Israel. We share initial ideas and concerns that were raised by the participating stakeholders, suggesting that developing a research network of committed individuals can be useful to attain this goal. We present the process as a possible approach that may be relevant to other countries striving to strengthen LE research and LE leadership (Byrne et al., 2018). We suggest this in homage to Prof. William Anthony’s (1993) deep legacy of recovery, and see this as the next step needed in order to rightly amplify the currently weakened voice of lived experience within mental health fields.

### Workshop Participants and Overview

The workshop focused on learning about diverse ways to involve people with lived experience across all stages of the research process. Examples were provided from the UK, showcasing coproduction of new service models and research studies that explored their acceptability and effectiveness, followed by responses of the participants.

The workshop participant group was comprised of thirty-two stakeholders from the field of mental health rehabilitation in Israel, including: service users, peer support workers, family members, clinicians, ministry of health personnel and academic researchers. Some participants had many years of experience in the field and held multiple roles as activists or served in key roles in the Ministry of Health. Others were C.E.Os or directors of services and senior mental health practitioners of various professions (e.g. psychiatric nurses, occupational therapists, psychologists and psychiatrists). During the introduction persons could self-identify as they chose to and reveal multiple roles such as being a clinician and mental health service user, a family member and an academic, etc. Ten participants explicitly self-presented on that day as having LE (three of them are the first three authors of this essay). It is likely that there were more persons with lived experience who chose to self-identify with their non-LE role in the session. For example, two of the authors in this essay (G.M & D.R) self-presented in the workshop by their ‘researcher’ identity, while both are also family members.

After introducing each other with an exercise designed to exemplify co-production in practice, Dr. Pinfold presented how public involvement in research and peer research can address key problems in mental health care drawing upon examples from the UK that involved lived experience researchers from the McPin Foundation. These included PARTNERS2 (PARTNERS2, 2020) and community mental health transformation (UK policy initiative), Community Navigators (complex depression and anxiety; Frerichs et al., 2020) and gameChange – a virtual reality research project (Bond et al., 2023). PARTNERS2 was a collaborative care program for people with severe mental illness seeking to bridge the gap in service provision between primary and secondary care, creating a new role ‘the care partner’ which adopted a person-centered, coaching focus and recovery led approach. The Community Navigators study was another example of the development of a new role – the community navigator (CN) – supporting people with complex depression and anxiety to address feelings of loneliness. Here the co-production group co-created the CN position, appointed and trained the staff to take up the new CN role, and were involved in qualitative evaluation of this roll out. Finally, gameChange was provided as an example of a virtual reality intervention supporting people with psychosis who experience social anxiety. The gameChange program was co-created with experts by experience so that all the scenarios were based upon common challenges. Lived experience researchers carried out a user-led study exploring people’s experiences of the therapy. These presentations offered vivid demonstrations about the unique benefits as well as complexities involved eliciting much thought and inspiration among workshop participants. In the final session of the day, participants shared their responses, ideas and questions.

In preparation of the current paper, the first author transcribed the workshop in real time and conducted an initial thematic analysis of the conversation. Together with the last author, they then refined the analyses which we bring here, articulated as principles and themes characteristic of a LE research workshop’. The second and fourth authors (with LE) and last author (researcher and family member) reviewed, commented and refined the themes presented. These reflect an approach that can actively involve people with lived experiences in research design and implementation in Israel in order to coproduce mental health policies and services that better meet the needs of people with LE (see Table 1). In a parallel manner to the workshop, this paper is also co-produced, authored by different stakeholders with and without LE. While the different voices are mostly embedded within the content, we chose to demonstrate different perspectives by adding two first-person depictions that will follow the overall responses and ideas.

**Table 1:** Principles and themes characteristic of a LE research workshop

Principles	Description
Co-production basics	- Work that generates trust and respect in teams - Valuing diverse talents and capabilities equally - Providing time and resources to everyone - Shared decision making processes
Closer engagement with communities	- Involving target audiences directly in project planning and execution - Incorporating community feedback to make research more community-centric
Applying Self-disclosure	- Emphasizing importance of context, gradual disclosure in research - Highlighting the importance of support systems within research teams - Exploring various aspects of personal exposure in research, from explicit to implicit disclosure, recognizing its contextual nature and potential evolution over time
Dealing with Bias and Reflexivity	- Acknowledging biases in research - Promoting reflexivity and open discussions within research teams - Considering intersectionality in research, recognizing the interconnected nature of social categories and how they influence experiences
Learning opportunities	- Promoting mutual learning through workshops, seminars, and online forums - Facilitating collaboration between researchers, practitioners, and a wider audience
Policy integration into practice	- Strengthening the connection between policy-making and daily practice - Suggesting initiatives like workshops or training days for professionals - Formulating policies that promote equality: providing economic support, educational opportunities, and projects that amplify the voices of underrepresented communities
Addressing Elitism and the power imbalance	- Encouraging projects that prioritize marginalized voices - Promoting unique and diverse perspectives in research
Challenging knowledge paradigms	- Supporting initiatives that encourage innovative thinking and contributions - Fostering collaborations across different disciplines - Address LE Research Value by emphasizing the need for more open and narrative approaches, as opposed to rigid, specific metrics
Empowering Stakeholders with Active involvement	- Creating work environments for real-time knowledge sharing between researchers and stakeholders - Involving regular planning meetings and collaborative participation in projects
Mentoring and Organizational Support	- Help individuals recognize and discuss the challenges they bring to their work - Develop mentoring programs, team-building, and a supportive work culture, contributing to staff well-being

### Stakeholders’ Responses and Ideas Regarding LE Research in the Israeli Context

Following the workshop, we held a discussion to gauge “where people are” in relation to taking forward a shared agenda around LE involvement in mental health research in Israel. Stakeholders raised critical questions (summarized in a list in Table 2) probing the value of LE, the ways in which such research can be effective in designing practices and policies, and ways to conduct research with greater LE impact and collaboration. Discussion focused on establishing a collaboration which will value the unique contribution of LE knowledge and LE leadership in research to enhance the quality of MH services.

**Table 2:** Participants’ questions regarding LE in community MH care

1	What constitutes as lived experienced?
2	How to develop the specialty of lived experience?
3	How personal experience research could enhance services?
4	What kind of questions can fund research & projects focusing on co-production?
5	How can we research issues of risk management, diversity, and inclusion?
6	How and when does research culminate into practical services?
7	What kind of research contributes to system change?
8	Where is the impact/value of research? Is there value in methodology & ethics?
9	What is the value of generalization in research with LE? Should it be a goal of research?
10	How do you measure the influence of lived experience on the research?
11	How should researchers with LE employ self-disclosure with interviewees?
12	How can employing a LE researcher improve a sense of ease amongst the people interviewed?
13	How to recruit people with LE and determine their skills and capacities to become part of research team?
14	What kind of support and supervision will the research team need?
15	How do you remain person centered in research that aspires to address large numbers, fidelity & standardization?
16	Should you incorporate LE research when working on development & implementation of a new service?
17	How to direct a team with LE research in a changing & flexible working environment?
18	How do we measure success of a new service in the context of LE guided research?

Some of the ideas that came up included: Using LE research to support recovery goals and “personal medicine” in services and policy. Researching LE within the continuum of services to promote comprehensive and coordinated care. And, employing experiential knowledge/LE inputs to guide development of new services and to study alternative models of care. Specifically, one peer activist—based on her own experience many years ago—shared her idea to develop a MH crisis-respite for mothers who experience postpartum depression where they can come with their baby. Another person suggested combining LE research with shared decision-making processes as a method to support and evaluate coproduction and recovery orientation in existing mental health settings. Family activists, in particular, voiced a need for more support from the system. They claimed that family members as caretakers provide a lot of the support needed to their loved ones mostly alone. As a result, they often experience a continued burden leading to family burnout. They called to involve family member perspectives in developing and evaluating family-related services. They viewed such an involvement as desired in and of itself in creating a more equal footing dialogue in service design and development. Finally, both stakeholders with LE and family members positively viewed the idea of conducting research in a co-productive manner with policymakers and professionals.

Interestingly, several participants pointed out that having a LE identity elicited sensitive issues both for themselves and in their relationships with others. For example, by having LE as well as other professional identities, some felt they end up feeling not fully belonging to either group of ‘service users’ nor ‘professionals’. Rather they felt a sense of being an “insider–outsider” is predominant when working or interacting with either professionals or service users. Others stated that quite a few individuals with LE in key roles in the MH system choose to remain undisclosed. Despite this, the commissioner of the rehabilitation Law claimed that having persons with LE in such senior positions in the government is still valuable and effective. Another participant claimed that subjectivity is not necessarily a barrier to objectivity: LE researchers can establish their arguments in scientific ways. These concerns and discussions reflected intrapersonal complexity and interpersonal stigma related to issues of self-disclosing LE in higher official ranks.

One of the participants (2nd author of this paper) with LE, who was in the past Chairman of The National Psychiatric Rehabilitation Council, mentioned that he often felt his participation in official meetings to be out of need for ‘consumer representation’, and his opinions framed as “emotionally biased” because of his MH history. At other times, when he voiced opinions regarding novel models of intervention, he got responses that framed his ideas as “pro-deinstitutionalization”, “radical” and/or “marginal”. For him, the participation in the workshop made him aware and appreciative of the importance of independent LE-expertise research as a key factor in promoting integration of recovery policy in the Israeli MH system. He stressed the importance of this initiative due to the complexity of the MH structure in Israel: on one hand, it is blessed with progressive laws and human rights sensitivity, and on the other hand, a prominent approach among policymakers still supports ultra conservative implementation. The Israeli MH system is still based on large and segregated psychiatric institutions (https://adva.org/wp-content/uploads/2023/05/Adva-MentalHealth-Budget2023-2024.pdf). In this kind of atmosphere, the roles of persons with LE evolve only if they embrace the psychiatric system as it is. That’s the reason for the high numbers of individuals with LE who work as consumers-providers and peer specialists in psychiatric hospitals, while the involvement of the consumer movement as a political factor is absent and the influence on psychiatric LE-research is limited. The workshop discussions about the idea of establishing a peer research council as a tool to strengthen the influence of consumers in research and policy left him with hope regarding implementations in the near future thus changing dynamics in the field. Mr. Oren claimed that in order to advance MH culture in Israel, more appreciation for LE in research, practice and policy making needs to develop carefully and efficiently.

### Dr. Pinfold’s Personal Response and Reflections on the Workshop

The invitation to share the expertise of the McPin Foundation with stakeholders in Israel considering setting up a lived experience research network was a unique experience. The workshop took place in early September 2023. When I conduct sessions about working with lived experience in mental health research, I can only do this because of the work my colleagues at McPin have delivered. We work collaboratively and recently we have been writing about our learning over 10 years including using lived experience expertise in the workplace (https://mcpin.org/projects-programmes/10-for-10/). My presentation in Israel was also based in part on my own experiences gained from being the PPI (patient and public involvement) lead in several large mental research studies. The examples I shared were chosen to fit with the themes of an upcoming conference in Tel Aviv exploring recovery, stigma and medication use.

Lived experience research is a developing area in mental health science. There is much to progress including unpacking what we mean by and approaches to intersectionality, identity-based disclosure, methods for generating experiential knowledge, epistemic injustice, dominant models from high income economies as well as inequalities and lack of diversity in the workforce. It was hard to decide what to cover, not knowing my audience beforehand. We adapted the workshop as we went, to ensure we could actively engage everyone in the room in a full conversation. This worked well. What I took away from the session was:

- The importance of inclusion – ensuring the network was built upon committed stakeholders that come from communities across Israel and included both people working in peer roles and those that were not bringing lived experience into their work roles. Power balances will need to be resolved and lived experience leadership prioritized.
- The importance of building upon peer support work within services – the expertise from peer leadership in Israel peer support services can support the development of lived experience in research.
- The need to build an infrastructure around lived experience research and public involvement in research starting with the funders and finding some allies within mental health services including academics and practitioners. People willing to share power and deliver collaborative working partnership with consumers and carers, as partners and not as research subjects. In the UK user-led studies were important for building the sector with sufficient resources. Partnerships with academics have also been vital – transforming research culture.

## Summary and Future Directions

Recovery oriented systems strive to incorporate lived experience in policies, practices, and research. LE has been increasingly recognized in various MH peer roles related to services in Israel. However, less emphasis was put on developing a systematic and integrated service-user/family member LE involvement in research. Here we described a workshop initiative, in which we began exploring the interest among multiple stakeholders to set up a lived-experience expertise coproducing research network in Israel.

The workshop elicited discussion about potential areas of focus for LE-led research as well as concerns regarding its applicability. The multiple themes that emerged in the workshop reflect potential research-initiative leads for service development and evaluation (e.g. personal medicine, continuum of care, gender-related novel models, family member service needs, co-production/shared decision evaluations). Interestingly, these topics correspond with Groot’s et al. (2022) review of 18 participatory research designs showing that participants tend to trans-diagnostic perspectives and prioritized different topics than experts. Power imbalance is known as a barrier to representation of service users’ and family members’ voice in research and development of health and mental health-related issues (Groot et al., 2022; LeBlanc & Kinsella, 2016). Similarly in our workshop, concerns regarding barriers in attaining a “patient perspective” were voiced around issues such as self-disclosure and (lack there-of) recognition of the value of knowledge from experience.

Finally, we are cognizant that a LE-led researcher council should aspire to represent diversity and be inclusive as much as possible. Inclusion of people with LE is recognized as central for achieving health service reform including commitments to human rights, social, and epistemic justice (Leblanc & Kinsella, 2016). The population in Israel is varied in religiosity, ethnicity, and geographical localities (peripheral vs. central). It has significantly grown and is expected to continue to grow especially in the Jewish and Arab communities (Weinreb & Shraberman, 2021). The development of a systemic structure with a diverse LE based coproducing research network can address the structural inequality and distribution of power in the mental health field by including stakeholders from different sections of society (religious/secular, diverse ethnic origins, and localities).

In the spirit of Bill Anthony’s legacy, we hope the integration of LE research into MH models of care, not only supports better recovery outcomes but also promotes a research culture that structurally reduces paternalism, lends itself to new means of communication among stakeholders, and truly adheres to recovery orientation.

## References

[CR1] Anthony, W. A. (1993). Recovery from mental illness: The guiding vision of the mental health service system in the 1990s. *Psychosocial Rehabilitation Journal,**16*(4), 11–23.

[CR2] Aviram, U., Ginath, Y., & Roe, D. (2012). Mental health reforms in Europe: Israel’s rehabilitation in the community of persons with mental disabilities law: Challenges and opportunities. *Psychiatric Services,**63*(2), 110–112. 22302325 10.1176/appi.ps.201100009

[CR3] Aviram, U., Lachman, M., & Ifergan, A. (2023). The Israeli law for the rehabilitation in the community of persons with psychiatric disabilities: Achievements and challenges. *Psychiatric Rehabilitation Journal,**46*(3), 185–195. 37707461 10.1037/prj0000582

[CR4] Banfield, M., Randall, R., O’Brien, M., Hope, S., Gulliver, A., Forbes, O., Morse, A. R., & Griffiths, K. (2018). Lived experience researchers partnering with consumers and carers to improve mental health research: Reflections from an Australian initiative. *International Journal of Mental Health Nursing,**27*(4), 1219–1229. 29847015 10.1111/inm.12482

[CR5] Beresford, P., & Rose, D. (2023). Decolonising global mental health: The role of mad studies. *Cambridge Prisms: Global Mental Health,**10*, e30. 37854430 10.1017/gmh.2023.21 PMC10579658

[CR6] Bond, J., Kenny, A., Pinfold, V., Couperthwaite, L., Kabir, T., Larkin, M., Beckley, A., Rosebrock, L., Lambe, S., Freeman, D., Waite, F., Robotham, D., gameChange Lived Experience Advisory Panel. (2023). A safe place to learn: peer research qualitative investigation of gamechange virtual reality therapy. *JMIR Serious Games,**11*(1), e38065. 36645707 10.2196/38065 PMC9947847

[CR7] Byrne, L., Stratford, A., & Davidson, L. (2018). The global need for lived experience leadership. *Psychiatric Rehabilitation Journal,**41*(1), 76. 29494198 10.1037/prj0000289

[CR8] Daass-Iraqi, S., Garber-Epstein, P., & Roe, D. (2021). Cultural adaptation of the illness management and recovery (IMR) intervention among Israeli Arabs. *Psychiatric Services*. 10.1176/appi.ps.202000175 33430648 10.1176/appi.ps.202000175

[CR9] Drake, R. E., Hogan, M. F., Slade, M., & Thornicroft, G. (2011). Editorial: commentary on Israel’s psychiatric rehabilitation law. *Israel Journal of Psychiatry and Related Sciences,**48*(4), 227–229. PMID: 22572085. 22572085

[CR10] Frerichs, J., Billings, J., Barber, N., Chhapia, A., Chipp, B., Shah, P., Shorten, A., Stefanidou, T., Johnson, S., Evans, B. L., & Pinfold, V. (2020). Influences on participation in a programme addressing loneliness among people with depression and anxiety: findings from the Community Navigator Study. *BMC Psychiatry,**20*(1), 1–15. 33243222 10.1186/s12888-020-02961-x PMC7689977

[CR11] Gelkopf, M., Lapid, L., Werbeloff, N., Levine, S. Z., Telem, A., Zisman-Ilani, Y., & Roe, D. (2016). A strengths-based case management service for people with serious mental illness in Israel: A randomized controlled trial. *Psychiatry Research,**241*, 182–189. 27179184 10.1016/j.psychres.2016.04.106

[CR12] Goldfarb, Y., Grayzman, A., Meir, L. G., Grundman, S. H., Rabinian, M., Lachman, M., Epstein, P. G., Ben-Dor, I. A., Naaman, A., Puschner, B., & Moran, G. S. (2024). UPSIDES mental health peer support in face of the COVID-19 pandemic: Actions and insights. *Community Mental Health Journal,**60*(1), 5–13. 36508063 10.1007/s10597-022-01030-9 PMC9743118

[CR13] Groot, B., Haveman, A., Buree, M., Zuijlen, R. V., Zuijlen, J. V., & Abma, T. (2022). What patients prioritize for research to improve their lives and how their priorities get dismissed again. *International Journal of Environmental Research and Public Health,**19*(4), 1927. 35206113 10.3390/ijerph19041927 PMC8871903

[CR14] Grundman, S. H., Edri, N., & Stanger Elran, R. (2021). From lived experience to experiential knowledge: A working model. *Mental Health and Social Inclusion,**25*(1), 23–31.

[CR15] Guta, A., Flicker, S., & Roche, B. (2013). Governing through community allegiance: A qualitative examination of peer research in community-based participatory research. *Critical Public Health,**23*(4), 432–451. 24273389 10.1080/09581596.2012.761675 PMC3827674

[CR16] Happell, B., & Roper, C. (2007). Consumer participation in mental health research: Articulating a model to guide practice. *Australasian Psychiatry,**15*(3), 237–241. 17516188 10.1080/10398560701320113

[CR17] Hasson-Ohayon, I., Roe, D., & Kravetz, S. (2007). A Randomized controlled trial of the effectiveness of the illness management and recovery program. *Psychiatric Services,**58*, 1461–1466. 17978257 10.1176/ps.2007.58.11.1461

[CR18] Hornik-Lurie, T., Shalev, A., Haknazar, L., Garber Epstein, P., Ziedenberg-Rehav, L., & Moran, G. S. (2018). Implementing recovery-oriented interventions with staff in a psychiatric hospital: A mixed-methods study. *Journal of Psychiatric and Mental Health Nursing,**25*(9–10), 569–581. 30411432 10.1111/jpm.12502

[CR19] Kraus, E., & Moran, G. S. (2019). When social work students meet workers with mental-health lived-experience: A case study. *Social Work Education*. 10.1080/02615479.2019.1593354

[CR20] Kraus, E., & Moran, G. S. (2023). What do BSW graduates need to know about mental health?: Educational insights derived from integrating service-users’, students’, and academic-educators’ perceptions. *Social Work Education*. 10.1080/02615479.2023.2229839

[CR21] Kraus, E., & Moran, G. S. (2023). The working mechanisms underpinning mental health experts by experience involvement in direct teaching: An abductive conceptual framework. *The British Journal of Social Work,**53*(8), 4002–4022. 10.1093/bjsw/bcad124

[CR22] LeBlanc, S., & Kinsella, E. (2016). Toward epistemic justice: A critically reflexive examination of ‘sanism’ and implications for knowledge generation. *Studies in Social Justice,**10*(1), 59–78. 10.26522/ssj.v10i1.1324

[CR23] Levy-Frank, I., Hasson-Ohayon, I., Kravetz, S., & Roe, D. (2011). Family psychoeducation and therapeutic alliance focused interventions for parents of a daughter or son with a severe mental illness. *Journal of Psychiatric Research,**189*(2), 173–179. 10.1016/j.psychres.2011.02.012 21482437

[CR24] Liberati, E., Richards, N., Parker, J., Willars, J., Scott, D., Boydell, N., Pinfold, V., Martin, G., Dixon-Woods, M., & Jones, P. (2021). Remote care for mental health: qualitative study with service consumers, carers and staff during the COVID-19 pandemic. *British Medical Journal Open,**11*(4), e049210. 10.1136/bmjopen-2021-049210 PMC8068948 33888531

[CR25] Mackay, T., Ahmed, N., Andleeb, H., Billsborough, J., Currie, R., Hazzard, R., Haider, F., Iqbal, N., Matthews, F., Mesarič, A., Parker, J., Pinfold, V., Richmond, L., Robotham, D., & Thompson, R. (2022). The evolution of community peer support values: reflections from three UK mental health project teams: The McPin peer support evaluation writing collaborative. *Advances in Mental Health,**20*(2), 157–169.

[CR26] Moran, G. S. (2017). A recovery-oriented peer provider (ROPP) work-role model and prototype measure. *American Journal of Psychiatric Rehabilitation,**20*(4), 346–368.

[CR27] Moran, G. S. (2018). The mental health consumer movement and peer providers in Israel. *Epidemiology and Psychiatric Sciences,**27*(5), 420–427. 10.1017/S2045796018000173 29656726 10.1017/S2045796018000173 PMC6999017

[CR28] Moran, G. S., Mashiach-Eizenberg, M., Roe, D., Berman, Y., Shalev, A., Kaplan, Z., & Epstein, P. G. (2014). Investigating the anatomy of the helping relationship in the context of psychiatric rehabilitation: The relation between working alliance, providers’ recovery competencies and personal recovery. *Psychiatry Research,**220*(1–2), 592–597. 25219616 10.1016/j.psychres.2014.08.004

[CR29] Moran, G. S., Westman, K., Weissberg, E., & Melamed, S. (2017). Perceived assistance in pursuing personal goals and personal recovery among mental health residents across housing services. *Psychiatry Research,**249*, 94–101. 28088068 10.1016/j.psychres.2017.01.013

[CR30] NIHR. (2019). Taking Stock – NIHR public involvement and engagement. Retrieved November 2023, from https://www.nihr.ac.uk/documents/taking-stock-nihr-public-involvement-and-engagement/20566

[CR31] Pagdon, S., & Jones, N. (2023). Psychosis outside the box: A user-led project to amplify the diversity and richness of experiences described as psychosis. *Psychiatric Services,**74*(7), 760–763. 36475822 10.1176/appi.ps.20220488

[CR32] PARTNERS2 Writing Collective. (2020). Exploring patient and public involvement (PPI) and co-production approaches in mental health research: Learning from the PARTNERS2 research programme. *Research Involvement and Engagement,**6*(2020), 1–11. 32974052 10.1186/s40900-020-00224-3 PMC7507647

[CR33] Richmond, L., Caton, N., Downs, J., Newton, A., Devereux-Fitzgerald, A., & Brooks, H. (2023). Creating positive experiences of involvement in mental health research. *The Lancet Psychiatry,**10*(3), 220–227. 36696910 10.1016/S2215-0366(22)00430-8

[CR34] Roe, D., Barniv-Bril, S., & Kravetz, S. (2012). Recovery in Israel: ensuring rights international review of psychiatry. *International Review of Psychiatry Issue on Recovery,**24*(1), 48–55. 10.3109/09540261.2011.652600 22385426

[CR35] Roe, D., Bril-Barniv, S., & Kravetz, S. (2012). Recovery in Israel: A legislative recovery response to the needs–rights paradox. *International Review of Psychiatry,**24*(1), 48–55. 22385426 10.3109/09540261.2011.652600

[CR36] Roe, D., Drake, R. E., & Slade, M. (2015). Routine outcome monitoring: An international endeavour. *International Review of Psychiatry,**27*(4), 257–326. 26271399 10.3109/09540261.2015.1070552

[CR37] Roe, D., Gelkopf, M., Gornemann, M., Baloush-Kleinman, V., & Shadmi, E. (2015). Implementing ROM in psychiatric rehabilitation services in Israel. *International Review of Psychiatry,**27*(4), 345–353. 25865480 10.3109/09540261.2015.1025722

[CR38] Roe, D., Hasson-Ohayon, I., Mashiach-Eizenberg, M., Derhy, O., Lysaker, P. H., & Yanos, P. T. (2014). Narrative enhancement and cognitive therapy (NECT) effectiveness: A quasi-experimental study. *Journal of Clinical Psychology,**70*(4), 303–312. 24114797 10.1002/jclp.22050 PMC3954406

[CR39] Roe, D., Slade, M., & Jones, N. (2022). The utility of patient-reported outcome measures in mental health. *World Psychiatry*. 10.1002/wps.20924 35015343 10.1002/wps.20924 PMC8751576

[CR40] Rose, D. (2021). Critical qualitative research on ‘madness’: Knowledge making and activism among those designated ‘mad.’ *Wellcome Open Research*. 10.12688/wellcomeopenres.16711.1 34368466 10.12688/wellcomeopenres.16711.1 PMC8314133

[CR41] Sweet, D., Byng, R., Webber, M., Enki, D. G., Porter, I., Larsen, J., Huxley, P., & Pinfold, V. (2018). Personal well-being networks, social capital and severe mental illness: exploratory study. *The British Journal of Psychiatry,**212*(5), 308–317. 28982657 10.1192/bjp.bp.117.203950

[CR42] Weinreb, A., & Shraberman, K. (2021). *Demographic trends in Israel: An overview* (pp. 271–285). Society, Economy and Policy.

